# Intellectual function evaluation of first generation immigrant children with sickle cell disease: the role of language and sociodemographic factors

**DOI:** 10.1186/1824-7288-39-36

**Published:** 2013-06-04

**Authors:** Maria Montanaro, Raffaella Colombatti, Marisa Pugliese, Camilla Migliozzi, Fabiana Zani, Maria Elena Guerzoni, Sheila Manoli, Renzo Manara, Giorgio Meneghetti, Patrizia Rampazzo, Francesca Cavalleri, Marco Giordan, Paolo Paolucci, Giuseppe Basso, Giovanni Palazzi, Laura Sainati

**Affiliations:** 1 Clinic of Pediatric Hematology-Oncology, Department of Pediatrics, Azienda Ospedaliera-University of Padova, Padova, Italy; 2 Clinic of Pediatric Hematology-Oncology, Department of Pediatrics, Azienda Ospedaliera-University of Modena, Modena, Italy; 3 Neuroradiology Unit, Azienda Ospedaliera-University of Padova, Padova, Italy; 4 Neurosonology Unit, Department of Neurological Sciences, Azienda Ospedaliera-University of Padova, Padova, Italy; 5 Neuroradiology Unit, Azienda Ospedaliera-University of Modena, Modena, Italy

## Abstract

**Background:**

Sickle Cell Disease (SCD) is the most common genetic disease worldwide. Neurological events are among the most worrisome clinical complications of SCD and are frequently accompanied by cognitive impairment. Intellectual function in SCD may vary according to genetic and environmental factors. Immigrant children with SCD are increasing at a global level and display specific health care needs. The aim of our multicenter study was to describe the intellectual function of first generation African immigrants with SCD and the influence of sociodemographic factors on its characteristics.

**Methods:**

The Wechsler Intelligence Scales were administered to evaluate broad intellectual functions in children with SCD and in age-matched healthy siblings. Patients’ clinical, socio-demographic, Magnetic Resonance Imaging (MRI) and Angiography (MRA) data were correlated to intellectual function scores.

**Results:**

68 children, mean age 8.95 years were evaluated. 72% spoke three languages, 21% two. FSIQ was <75 in 25% of the children. Mean VIQ was lower than PIQ in 75%. Mean verbal subtest scores were lower than performance scores. Female gender, number of languages spoken at home and mother’s employment were associated with single subtest performances (p < 0.05). MRA was abnormal in 73.4% and MRI in 35.9%. No significant correlation was established between silent lesions and intellectual function, even if patients with lesions performed worse. Fifteen siblings performed better than patients on cognitive domains, including language (p < 0.05).

**Conclusions:**

Immigrant bilingual children with SCD seem to display a rate of cognitive impairment similar to their monolingual counterparts but a more pronounced and precocious onset of language difficulties. Adjunctive tests need to be considered in this group of patients to better define their specific deficits.

## Introduction

Sickle Cell Disease (SCD) is the most common genetic disease worldwide and is recognized by the World Health Organization (WHO) and the United Nations (UN) as a global public health problem [1-3]. Despite being a hemoglobinopathy due to a single gene mutation, SCD shows extreme phenotypic variability among individuals and among populations [3,4]. Both environmental (climate, socio-cultural and demographic influences, language) and genetic factors are likely to contribute to most manifestations of SCD that develops with different patterns in various ethnic groups and individuals [4,5]. Neurological events (stroke, silent infarcts and transient ischemic attacks) are among the most worrisome clinical complications of SCD and are frequently accompanied by cognitive impairment. Most studies assessing intellectual function in SCD patients have been performed in the United States (USA) [6-8] few were replicated in Western Europe [9-11] where the health and education al systems are different and only one in Africa, were the majority of patients with SCD live [12]. It has been suggested that cerebrovascular complications of SCD might occur with different prevalence in Africans compared to Caucasians or African-Americans [13,14]. Similarly, different patterns of impairment in intellectual function are beginning to emerge among SCD children with different ethnic backgrounds or living in different countries [6-12]. To our knowledge, no information is available on intellectual function evaluation of immigrant children with SCD that speak different languages from the one of the hosting country, like English and French-speaking patients in Southern Europe or Hispanic immigrants in the USA. Bilingual or multilingual immigrant children with SCD represent a population in which the evaluation of cognitive impairment represents a challenge. Bilingualism itself represents a risk for developing specific educational problems [15] and failing in standardized intellectual function test [16].

In Italy the majority of children with SCD are immigrants coming from African countries, mainly English or French-speaking countries [17]. Immigrants present socio-demographic characteristics that have an effect on the management of chronic illnesses and might impact the evaluation of cognitive impairment.

Objectives of our study were to: 1.Describe the intellectual function of first generation immigrant children with SCD 2. Determine the role of socio-demographic and linguistic factors on intellectual function 3. Identify possible areas of intervention for cognitive and educational rehabilitation in this particular population.

## Methods

### Setting

The Pediatric Hematology-Oncology Unit of the Azienda Ospedaliera-Università of Padova and Modena, are located in two cities in Northern Italy where immigrants represent 10-14% of the population and newborns from immigrant couples represent 25-30% of the newborns [18].

Children with SCD accessing the two centers are offered comprehensive care with particular attention to the needs of immigrant patients since 2006, as previously described [17]. Since 2009 intellectual function evaluation and neuroimaging studies are routinely performed as part of comprehensive care. Clinical, social and demographic information on all patients are prospectively and systematically collected in a clinical database.

### Intellectual function evaluation

Intellectual function evaluation, conducted by licensed psychologists, was performed using the Wechsler scales. Tests were administered according to a standardized protocol, defined in two preparatory meetings in order to minimize disparities between Padova and Modena. Intellectual function evaluation was performed on the same day of a routine hematology visit, with children in steady state. It took place in a separate and quiet room within the Pediatric Hematology-Oncology Units. The tests were administered always in the same order and were performed in Italian. Administration of the entire battery (including breaks) required 1 ½ hour-2 hours. Intellectual function evaluation was performed also in age matched siblings that routinely came to clinic together with the patients.

The Wechsler Intelligence Scales (Italian standardization) [19,20], standard psychometric tests of general intellectual development, were chosen according to the child’s age: the Wechsler Intelligence Scale for Children-III (WISC-III) for children aged 6.7-16 years, the Wechsler Preschool Scale Intelligence (WPSSI) for those aged 4–6.6 years.

The following age-adjusted scores were reported: Full-scale IQ (FsIQ), Verbal IQ (VIQ) and Performance IQ (PIQ). The WISC-III battery of tests included also six Verbal and six Performance Subtest and the following composite factor scores, allowing a broad characterization of specific cognitive domains: Verbal Comprehension (VC), Perceptual Organization (PO), Freedom from Distractibility (FD) and Processing Speed (PS). The WPSSI‘s battery of tests also includes five Verbal and five Performance Subtest.

Intellectual function data were centrally collected in Padova and were rescored to ensure that all data were properly scored; subsequently they were entered into an excel database. Neuropsychologists and neuroradiologists were blinded to clinical findings and to each other’s results.

The IRB of the Azienda Ospedaliera-Università of Padova and Modena approved the study protocol. Written informed consent was obtained from parents.

### Magnetic Resonance Imaging (MRI) and Magnetic Resonance Angiography (MRA)

All MRI studies were performed on 1.5 Tesla scanner (Achieva, Philips, Best The Netherlands).

The study protocol included Fluid Attenuated Inversion Recovery (FLAIR; TR: 10000ms, effective TE: 140 ms; TI: 2100 ms; matrix 256×320) and diffusion weighted imaging (DWI; TR: 3948 ms, TE: 96 ms, matrix: 192×153, b-value: 1000 s/mm2) with identical position, field of view (230 mm), slice thickness (5 mm) and gap (0.5 mm). Volume of ischemic lesions was calculated after manually drawing the signal abnormalities on FLAIR images [∑ Area lesions × (slice thickness + interslice gap)] using a dedicated software (MedStation^®^). Silent infarctsl (SI) were defined as an MRI signal abnormality of at least 3 mm in one direction and visible on two views on FLAIR T2-weighted images in a patient with normal neurological examination [21]. MRA was obtained using a three-dimensional time of flight technique (flip angle: 20, repetition time: 25 ms, echo time: 6.9 ms, field of view: 160 × 160 mm, slice thickness: 0.5 mm, matrix: 320 × 183). Severity of occlusive changes of the internal carotid artery siphon (s-ICA), of the main segments of the anterior (A1-, A2-ACA), middle (M1-, M2-MCA) and posterior (P1-, P2-PCA) cerebral arteries was scored as follows: 0 (normal), 1 (mild stenosis), 2 (severe stenosis), 3 (occlusion) [22]. All images were centrally reviewed by two experienced neuroradiologists.

### Statistical analyses

Statistical analyses were performed using the R program (http://www.r-project.org). To compare children with SCD from Padova and Modena on subtest scores and socio-demographic characteristics we used t-tests for quantitative variables and Chi-square tests for categorical variables. Regression analysis was used to explore the relationship between cognitive scores and socio-demographic aspects, MRI and MRA findings. To choose an appropriate model we used a “backward” stepwise model selection by AIC (Akaike Information Criterion). The p-values were corrected to control the family-wise error rate with Holm’s method and were considered significant if < 0.05.

## Results

### Socio-demographic characteristics

Between October 2009 and June 2011, 68 children (54 HbS/HbS, 3 HbS/Betathalassemia, 11 HbS/HbC), mean age 8.95 years (range: 4–15 years) were evaluated, 47 in Padova and 21 in Modena. Males were 32, Females 36. The majority (67/68) were first generation immigrants (47 from African English-speaking countries, 14 from African French-speaking countries, 3 from Portuguese-speaking countries, 3 from Albania); 4 children were adopted. 66.2% were born in Italy. Mean years of living in Italy was 4.86 yrs (range 1–15 years) if not born in Italy. 72% of the children spoke three languages, 21% two and 7% only Italian, but all children spoke fluent Italian and used this language for day-by-day interactions with friends and teachers. Communication during routine health visits had to be conducted by the physician in English or French for 60% of the families because parents had a better comprehension in their mother-tongue language rather than in Italian.

The majority of the parents (60%) had a high school diploma obtained in the country of origin. At the time of intellectual function evaluation all families were legal immigrants; 86% of the fathers and 54% of mothers were employed. Parents’ socio-demographic characteristics are shown in Table 1. No differences in sociodemographic characteristics were observed between children living in Padova and Modena (p > 0.05).

**Table 1:** **Parents**’ **socio**-**demographic characteristics**

**Variable**				
**Mean years living in Italy**	**9.65 years (range 2–20) SD 4.56**			
**Country of origin (N, %)**				
English-speaking African countries (Nigeria, Ghana)	47	69%		
French-speaking African countries (Senegal, Togo Guinea, Camerun, Congo, Burkinafaso)	14	22%		
Others (Portorico, Brazil, Albania)	6	7.5%		
Italy	1	1.5%		
**Parental employment (N, %)**	**father**		** mother**	
Worker in a factory	45	66.2%	17	25.0%
Office worker	8	11.8%	20	29.4%
Job professional	2	2 .9%	0	
Housewife	0		21	30.9%
Not employed	6	8.8%	6	8.8%
Not specified	7	10.3%	4	5.9%

### Clinical characteristics and Magnetic Resonance

None of the children had been born preterm or had experienced a clinical stroke. Neurological evaluation was normal in all patients. Twelve children (17.6%) were on Hydroxiurea (HC) for recurrent Acute Chest Syndromes or Vaso-occlusive crisis and 11/68 (8.3%) were on chronic transfusion, seven due to abnormal Transcranial Doppler. MRI and MRA were performed in 64/68 (94%). MRA was abnormal in 47/64 (73.4%) and MRI in 23/64 (35.9%). MRI data and their relationship with FsIQ, VIQ and PIQ are reported in Table 2.

**Table 2:** **Correlations between Magnetic Resonance Imaging** (**MRI**) **and FsIQ**, **VIQ**, **PIQ**

	**No lesions**	**Lesion burden <500 mm3**	**Lesion burden >500 mm3**	**p-value**
***WISC-III***				
N Patients	29	11	6	0.00
Mean Age (years)	9.95	11.41	9.68	0.41
FsIQ	89.53	88.54	72.50	0.26
VIQ	85.89	88.72	70	0.26
PIQ	94.41	91.18	81.83	0.41
***WIPSSI***				
N Patients	12	3	3	0.07
Mean Age (years)	5.63	5.76	4.67	0.35
FsIQ	86.08	87.66	109.33	0.24
VIQ	80.83	78.33	102.66	0.33
PIQ	94.66	100	114.33	0.35

### Intellectual function evaluation

**Figure 1:**
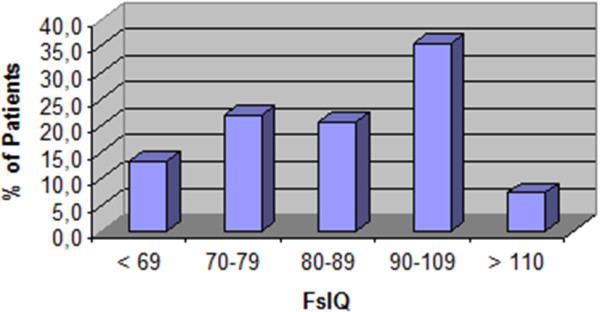
**Distribution of Full**-**scale IQ scores in children with SCD.**

All families accepted to perform the intellectual function evaluation. 25% of children were in kinder-garden, 50% in primary school, 18% middle school, 7% high school. Some children (5/68) had already been diagnosed with intellectual function or behavioral problems and were receiving educational tuition during classes and 3 more were in the process of receiving it. FSIQ was below 75 in 25% of the children. VIQ was lower than PIQ in 46 patients (76.5%). Detailed distribution of FsIQ is reported in Figure 1 while results of WISC-III and WPSSI are given in Table 3.

**Table 3:** **Detailed results of the Wechsler scales and comparison between older children** (**WISC**-**III**) **and younger children** (**WPPSI**)

**Scores**	**WISC-III (n=49)**		**WPPSI (n=19)**		**p value**
	**Mean**	**SD**	**Mean**	**SD**	
**IQ**					
Full-scale IQ	86.9	16.3	89.6	15.5	N.S.
Verbal IQ	84.5	17.3	83.8	16.7	N.S.
Performance IQ	91.7	15.8	98	16.2	N.S.
**Verbal subtests**					
Information/General Culture	7.7	3.2	8.0	2.7	N.S
Similarities	8.8	3.2	9.0	3.1	N.S.
Arithmetic	6.7	2.9	8.0	3.5	N.S.
Vocabulary	7.5	3.0	7.4	2.6	N.S.
Comprehension/General Comprehension	7.6	3.9	4.9	3.3	N.S.
Digit Span/Phrases	8.2	3.5	N.E.		
**Performance subtests**					
Picture Completion	10.6	4.1	11.1	3.1	N.S.
Coding/Animal House	7.1	3.3	9.6	3.3	N.S.
Picture Arrangement/Retest Animal House	8.4	2.6	N.E.		
Block Design	9.1	2.8	8.7	3.1	N.S.
Object Assembly/ Geometric Design	8.9	2.5	9.7	3.3	N.S.
Symbol search	8.5	3.4	N.E.		
Mazes	9.6	3.8	8.7	3.1	N.S.

Abbreviations: *N.S.* Not Significant, *N.E* Not Evaluated.

WISC-III VC and FD factor scores were lower than the reference values, indicating difficulties in general language skills and attention, concentration, working memory (Table 4).

**Table 4:** **Mean WISC**-**III factor scores of children with SCD compared to normal reference values**

**Factor scores**	**Mean**	**SD**	**Normal reference values**
**Verbal comprehension**	86	20.6	>90
**Perceptual organization**	95	20.5	>90
**Freedom from Distractibility**	85	17.0	>90
**Processing speed**	92	19.6	>90

Clinical observation of behavior was routinely performed during cognitive assessment only in Padova. The majority of the 47 patients (83%) displayed behavioral problems: slowness in performing the test (n.19), low self-esteem (n.18), performance anxiety (n.10), difficulties in understanding the tasks (n.10), impulsivity (n.10), oppositional behavior (n.5), attention deficiency (n.4), and hyperactivity (n.4).

Families were invited to discuss the results: all families accepted to discuss them and asked for support in dealing with school teachers.

No differences were observed in mean FsIQ, VIQ, PIQ and mean subtests scores for children on HC, on chronic transfusion or without specific treatment. When analyzed separately, FSIQ, VIQ, PIQ and subtests of HbSS/HbSbetathalassemia patients were similar to the ones of HbS/HbC patients.

### Socio-demographic determinants of intellectual function

Children born in Italy scored worse on VIQ WISC-III (*p 0*.*049*, coeff. -11.97, SE 5.58), Comprehension subtest (p 0.029, coeff. -4.14, SE 1.33) and VC factor (p 0.045, coeff. -32.45, SE 9.55), indicating poorer verbal abilities than children born abroad. Children born in Italy were younger than children born abroad (8.33 vs 10.17 years).

Female gender, number of languages spoken at home and parent’s level of employment was associated with single subtest performances. Female gender was associated with better performances in Comprehension WISC-III subtest (p 0.038, coeff. -3.80, SE 1.28), indicating greater practical reasoning ability. Speaking two languages rather than three was associated with better scores on Picture Arrangement WISC-III subtest (*p 0*.*013*, coeff. 6.23, SE 1.58). Children of mothers working as “employee” displayed better scores on Coding WISC-III subtest (*p 0*.*007*, coeff. = 7.0, SE = 2.04) and PS factor (p 0.039, coeff. = 33.84, SE = 11.19) while having a “not employed” father was associated with lower scores on Picture Arrangement (p 0.028, coeff. = −4.40, SE = 1.23) and Similarities WISC-III subtests (p 0.006, coeff. = −7.36, SE = 1.63).

Although no significant differences were revealed between younger and older children (Table 3), FsIQ and PIQ tended to decrease with age, while VIQ remained similar across ages. Younger age was associated with better scores on most of the performance subtest, except for Block Design and for Mazes, indicating poorer visual perceptual skill and poorer planning ability in following simple directions.

No correlation was found between FsIQ, VIQ, PIQ and the presence of silent infarcts on MRI or stenosis on MRA (p value > 0.5 for all). When considering lesion volume, children with MRI lesion volume >500 mm3 scored worse than children with lesions <500 mm3 and children with no lesions.

Patient’s mean intellectual function scores significantly were lower than the ones of 15 healthy siblings (mean age 10.06 years): siblings displayed better results on FsIQ (93 vs. 86.9), VIQ (92 vs. 84.5), PIQ (95 vs. 91.7) and all subtests (p < 0.05).

## Discussion

Our study shows a rate of intellectual function impairment higher than the one reported in USA surveys (25% vs 9%) despite a similar rate of silent infarcts (30-36%). Moreover, our cohort of immigrant children with SCD seems to display a specific pattern of intellectual function impairment, associated with more compromised language abilities compared to similar socio-demographic groups.

Unlike other reported series, our multicenter population did not present any overt stroke. This data is lower than other reported series. It has been suggested that stroke prevalence might be different in African patients compared to African American ones. In fact the recent, but still limited data available from African cohorts, seems to confirm this hypothesis, showing a prevalence of stroke that goes from 0% to 8.4% [23-25]. Moreover, since 2007 all our children were performing Transcranial Doppler screening for stroke prevention and at risk children were enrolled in a transfusion program. Both factors could have contributed to our low stroke rate. Nevertheless, we had a high rate of abnormal MRI (35.9%), similar to the one recently reported in American and European cohorts [9,10,26]. Our population displayed 25% of children with cognitive impairment (FsIQ < 75), higher than the rate reported in African Americans in the USA [8], but similar to the one described in immigrant children with SCD in France and the Netherlands [9,10] and much lower than the 37.5% reported in African children with SCD in Cameroon [12]. Cultural factors deriving from the different origin of the patient population and socioeconomic determinants linked to their immigrant status could be crucial in determining these differences between American and European cohorts.

Our mean FsIQ and PIQ are similar to the ones observed in children with SCD in the USA and Europe as shown in Table 5[6,8-11,26-32]. Differently from all other studies except one [6], the mean VIQ of our population is lower than the mean PIQ and 76% of our patients had VIQ lower than PIQ, revealing poorer language ability compared to general logic and suggesting an impairment of language and verbal functions in our population. This is further confirmed by the low mean in the verbal subtests and in the WISC-III factor scores. With the exception of the study by Armstrong et al. [6] who reported a poorer performance on FSIQ, VIQ and PIQ in children whose families spoke more than one language at home (English plus another language), our study is the first to show a poorer performance on language domains compared to general logic in children with SCD not having experienced an overt stroke. Moreover, our analysis reveals low VIQ and verbal subtest scores also in younger children who perform even worse than older ones, suggesting the role of precocious factors in determining language impairment in our population. Language processing deficits in all three dimensions of semantic, syntactic and phonologic processing have indeed been demonstrated in school-age children with SCD [11,29,32,33]. It has been hypothesized that lower scores on VIQ in school age children might depend from school absenteeism [33] and therefore be an indirect effect rather than a direct manifestation of the disease itself. Our data and the report of language impairment in African immigrant infants with SCD in the UK question this hypothesis and suggest a more complex etiology of language disabilities [29]. Even if no clear correlation was demonstrated between VIQ or verbal subtest scores and socio-demographic factors, speaking two languages –instead of three- and having an employed mother –interacting with the Italian society- allowed better results in some subtests. Moreover, children born in Italy, but with families that have only recently moved to our country, performed worse than older children born abroad but living in Italy since long ago. One possibility could be that immigrant children and families who lived in Western countries only for few years may display lower scores due to the unfamiliarity with the test material [12]. Alternatively, we have to consider that almost all our patients were multilingual, 70% routinely speaking three languages at home and the remaining at least two. Bilingualism and multilingualism could adversely influence language processing in children with SCD and the role of bilingualism must be underscored while performing intellectual function evaluation in first generation immigrants whose home language is different from the one of the hosting country. In fact, bilingual children take longer to acquire higher-order language proficiency [34]. While their fluency may be adequate for day-to-day interactions, it falls short of the higher-order fluency required for cognitive processing in the “context reduced” situation as the intellectual function evaluation [16,35]. This may at least in part help to explain why bilingual children with SCD may perform worse on standardized test assessing language domains than their monolingual counterparts.

**Table 5:** **Comparison of Mean FsIQ**, **VIQ**, **PIQ in different populations with sickle cell disease in Europe and USA**

	**Age years**	**% with FsIQ<75**	**FSIQ**	**VIQ**	**PIQ**	**% Immigrant**	**% mother-tongue language of hosting country**
USA	8.5	NR	70.8-82.8-90*	72.1-79.9-88.8*	74.1-88.1-92.9*	NR	NR but IQ lower in children speaking two languages at home
Armstrong 1996							
USA	13.5	9	77.3-81.1-86.7*	79.9-77.1-85.3*	77.3-81.1-86.7*	NR	NR
Wang 2001							
USA	9.5	NR	78.6-81.1**	74.1-84.6**	77.5-78.9**	NR	NR
Steen 2003							
USA	9.75	NR	75.05-81.91-81.67*	79.59-88.73-85.60*	74.73-77.73-80.80*	NR	NR
Brown 2000							
USA	8.2	NR	72.7-85-90.2*	74.2-84.6-93*	75.5-83.3-89*	NR	NR
Thompson 2003							
USA	10	NR	87.15-90.33-91.22§	89.56-94.86-92§	86.9-87.93-91.96§	NR	100
Kral MC 2003							
UK	8.73-11.58	NR	67.6-79-86.03*	73.6-85.25-89.07*	67.80-79.75-85.57*	NR	NR
Watkins 1998							
France	5-15	30.7	83.9-82.6-86.6**	90.4-85.8-93.3**	80.6-83.5-82.3	NR	NR but performed in children living in France > 2 yrs
Bernaudin 2000							
UK	infants	NR	82.5-87.4**	BIN study early language development in infants	BIN measures of early language development in infants	100	BIN measures of All families spoke English, and in the majority of cases, infants were also exposed to a non-English
Hogan 2006							
The Netherlands			79-80**	80-82**	80-77**	NR	NR
Hijmans 2010							
The Netherlands	12.2	30	80	83	79	100	46 (immigrants from Netherland Antilles)
Hijmans 2011							
This paper	8.9	25	86.9	84.5	91.7	100	1

*Clinical stroke Silent infarct on MRI- Normal MRI; **Silent Infarct on MRI Normal MRI; § Abnormal TCD-Conditional TCD-Normal TCD, *NR* not reported.

The better results obtained by siblings on intellectual function tests, including VIQ and verbal subtests, suggest that the lower mean VIQ and verbal subtest scores displayed by patients could be more disease-related rather than due to a lack of familiarity with the Italian language or to specific socio-demographic characteristics. Increasing patient and sibling-control sample size to compare bilingual affected and non affected children and analyzing clinical markers of disease severity might aid in determining the specific weight of multilingualism and socio-cultural factors on language disabilities.

In our study no significant correlation was established on multivariate analysis between silent infarcts and intellectual function evaluation, including VIQ, even if patients with lesions performed worse according to lesion burden. Hijmans and collegues in 2011 [11] also failed to demonstrate correlation between neurocognitive scores and silent infarcts in a population of mainly immigrant children coming from the Netherland Antilles. We believe that the presence of multiple cofounding factors (linguistical, socio-cultural) could be responsible for this lack of significance in our populations or, alternatively, such cofactors could be a more important determinant of cognitive function than other biological markers in a population of immigrant children. Moreover, the battery of tests that has been used up to now for children with SCD could be less appropriate for our population of bilingual immigrant children and further test need to be considered. Previous reports have shown that intellectual function performance is not adequately explained by findings on standard neuroimaging studies and further research is necessary in this field [23,36].

The early age of onset of language difficulties in immigrant bilingual children with SCD, regardless of its causes, suggests the routine and baseline use of specific language evaluation tools in this group of patients to detect precocious deficits and provide prompt rehabilitation.

Despite the new findings of our study in describing a group of patients with SCD having specific health-care and intellectual function needs, our study has several limits. First, the population size is limited, although bigger than many reported studies and with the advantage of being multicenter survey. Sample size might have prevented the emergence of definitive results. Second, the sample of young children is small therefore the school naïve group should be enhanced in order to better define the disease related profile of cognitive impairment in patients having experienced less chronic disease effects. Third, correlation with MRI was performed only with lesion burden; considering lesion site and functional MRI could aid in determining possible correlations between language disabilities and specific lesion patterns. Moreover, a comparison between European, American and African cohorts on intellectual function and clinical data could differentiate the effects of organic from psychosocial processes of intellectual functioning.

## Conclusions

Our data demonstrate that routine cognitive assessment is feasible as part of comprehensive care for high risk vulnerable groups such as immigrants. Immigrant patients are willing to receive psychosocial support and this can be an aid to social integration in the new country. African immigrant bilingual children with SCD seem to display a similar rate of cognitive impairment compared to monolingual immigrant children, but with lower scores in the verbal domains. Routine and baseline use of specific language evaluation tools are suggested as part of neurocognitive evaluation in this group of patients to detect precocious deficits and provide prompt rehabilitation.

## Competing interests

All authors declare that they have no competing interests.

## Authors’ contribution

MM, MP, CM, FZ, SM designed the study and conducted the intellectual evaluation; MEG, GP, RC and LS collected and interpreted clinical data; RM, GM, PR and FC performed neuroradiological analyses, collected and interpreted the data; MM and RC wrote the manuscript; LS, GP, PP and GB reviewed the Manuscript. All authors read and approved the final manuscript.
